# Cognitive Capacity Genome-Wide Polygenic Scores Identify Individuals with Slower Cognitive Decline in Aging

**DOI:** 10.3390/genes13081320

**Published:** 2022-07-24

**Authors:** Yoonjung Yoonie Joo, Jiook Cha, Jeremy Freese, M. Geoffrey Hayes

**Affiliations:** 1 Division of Endocrinology, Metabolism, and Molecular Medicine, Department of Medicine, Northwestern University Feinberg School of Medicine, Chicago, IL 60611, USA; 2 Department of Psychology, College of Social Sciences, Seoul National University, Seoul 08825, Korea; connectome@snu.ac.kr; 3 Institute of Data Science, Korea University, Seoul 02841, Korea; 4 AI Institute, Seoul National University, Seoul 08825, Korea; 5 Department of Brain and Cognitive Sciences, College of Natural Sciences, Seoul National University, Seoul 08825, Korea; 6 Department of Sociology, School of Humanities and Sciences, Stanford University, Palo Alto, CA 94305, USA; jfreese@stanford.edu; 7 Center for Genetic Medicine, Northwestern University Feinberg School of Medicine, Chicago, IL 60611, USA; 8 Department of Anthropology, Northwestern University, Evanston, IL 60208, USA

**Keywords:** cognitive genetics, aging genetics, phenome-wide association study, genome-wide polygenic score, sociogenomics

## Abstract

The genetic protective factors for cognitive decline in aging remain unknown. Predicting an individual’s rate of cognitive decline—or with better cognitive resilience—using genetics will allow personalized intervention for cognitive enhancement and the optimal selection of target samples in clinical trials. Here, using genome-wide polygenic scores (GPS) of cognitive capacity as the genomic indicators for variations of human intelligence, we analyzed the 18-year records of cognitive and behavioral data of 8511 European-ancestry adults from the Wisconsin Longitudinal Study (WLS), specifically focusing on the cognitive assessments that were repeatedly administered to the participants with their average ages of 64.5 and 71.5. We identified a significant interaction effect between age and cognitive capacity GPS, which indicated that a higher cognitive capacity GPS significantly correlated with a slower cognitive decline in the domain of immediate memory recall (β = 1.86 × 10^−1^, *p*-value = 1.79 × 10^−3^). The additional phenome-wide analyses identified several associations between cognitive capacity GPSs and cognitive/behavioral phenotypes, such as *similarities* task (β = 1.36, 95% CI = (1.22, 1.51), *p*-value = 3.59 × 10^−74^), *number series* task (β = 0.94, 95% CI = (0.85, 1.04), *p*-value = 2.55 × 10^−78^), *IQ scores* (β = 1.42, 95% CI = (1.32, 1.51), *p*-value = 7.74 × 10^−179^), *high school classrank* (β = 1.86, 95% CI = (1.69, 2.02), *p*-value = 3.07 × 10^−101^), Openness from the BIG 5 personality factor (*p*-value = 2.19 × 10^−14^, β = 0.57, 95% CI = (0.42, 0.71)), and leisure activity of reading books (β = 0.50, 95% CI = (0.40, 0.60), *p*-value = 2.03 × 10^−21^), attending cultural events, such as concerts, plays, or museums (β = 0.60, 95% CI = (0.49, 0.72), *p*-value = 2.06 × 10^−23^), and watching TV (β = −0.48, 95% CI = (−0.59, −0.37), *p*-value = 4.16 × 10^−18^). As the first phenome-wide analysis of cognitive and behavioral phenotypes, this study presents the novel genetic protective effects of cognitive ability on the decline of memory recall in an aging population.

## 1. Introduction

The magnitudes of cognitive decline in aging, a major health concern in contemporary society, differ substantially across individuals [1,2]. Existing literature suggests that the heterogeneity of cognitive decline is partially owing to the fact that some adults are more resilient to neuropathological changes than others [3,4,5]. Cognitive resilience is conceptualized as an individual’s capacity to overcome negative effects or stress on cognitive functioning despite aging or neuropathologic changes [6,7]. Diverse factors have been hypothesized to explain cognitive resilience, including brain structural features, genetic factors, and personality attributes acquired over the lifespan that offset the negative effects (i.e., cognitive decline) of brain aging, insult, or pathologies [8,9]. Elucidating the underlying mechanisms that may differentiate and identify aging adults with high or low cognitive resilience is essential to inform the homogeneity of adults in clinical trials for risk stratification and providing preventive intervention.

Studying cognitive resilience in the elderly is of special interest because the finding may provide insights into maintaining good cognition and “aging well” without developing dementia due to Alzheimer’s disease or other causes. However, while studies have reported the genetic risk factors of accelerated cognitive decline among individuals with dementia [10,11,12], we know very little about the genetic protective factors against cognitive decline that are present in the normal aging population. Recent studies have identified several genes and proteins that are assumed to be associated with cognitive resilience [9,13,14], but no study has yet aggregated multiple genetic variants into a single score that may summarize individual-specific indices of cognitive capacity and their impacts on cognitive resilience and other related attributes. In addition, most studies have not assessed the inherited genetic profile of resilient people, nor have they associated these genetic factors with the rate of cognitive decline or other behavioral life course outcomes, which may help us understand the genetic backgrounds of resilient and non-resilient people, along with how variations in genetic profile affect life course alterations other than cognition. Genome-wide polygenic scores (GPS) leverage the fact that most human traits are the result of the aggregated influence of many genetic variants, both common and rare [15,16,17]. By aggregating the minuscule effects of millions of genetic variants into a single score, GPS allows researchers to stratify individuals by their genomic propensity for a particular trait and select individuals with extremely high or low GPS for further research. The recent large genome-wide association studies (GWAS) of *educational attainment*, an often-used proxy phenotype for human intelligence, identified 1271 independent autosomal loci reaching genome-wide significance [18]. These findings suggest that several biological pathways related to brain development or neuron-to-neuron communication contribute to human intelligence. While the GWAS revealed many genetic variants associated with cognitive capacity phenotypes (such as cognitive performance, math ability, and highest math class taken) [18,19,20,21,22,23,24,25], the genomic contribution to specific cognitive domains remains unknown, as does their relationship to cognitive changes with aging.

Since general cognitive ability is known to be highly heritable (50–70%) and polygenic [26,27], we utilized GPS to account for the genome-wide factors underlying cognitive capacity and its changes with aging [21,24,28,29]. We leveraged the comprehensive phenotype information of a 50+ year social longitudinal database for phenome-wide association studies (PheWAS). The Wisconsin Longitudinal Study (WLS), the longest-running social longitudinal study in the United States [30,31], encompasses a detailed and broad lifelog of cognition, personality, financial, health, and socioeconomic status. The surveys have been repeatedly administered the same cognitive ability tests with the time interval of ~10 years in their latest survey rounds, as well as collected the genotype data of the participants, which creates a deep genotype-phenotype catalog of an individual’s cognitive and behavioral traits over their adult lives (Files S1).

Herein, we hypothesize that the polygenic influence of the cognitive capacity can explain certain patterns of cognitive abilities and the rate of their decline during aging and other socio-behavioral phenotypes that might be affected by the genetics of cognitive abilities. We tested the associations between the longitudinal observations of individual cognitive/behavioral phenomes and the GPSs of four different cognitive phenotypes (*educational attainment*, cognitive performance, math ability, and *highest math class taken*), focusing on the secular changes in cognitive test scores. The approach was designed to systematically address the following research questions: first, whether a particular cognitive domain was more impacted by polygenic influence than other cognitive domains; secondly, whether individuals with different GPSs showed different patterns of cognitive decline during aging, which suggests whether attributes of cognitive resilience are genetically inherited; and, thirdly, the extent to which the phenotypic variances of the behavioral/personality attributes could be explained by the genetic liability of the cognitive capacities which may help us understand how the different genetic profiles of resilient people affect their life course alterations other than cognition. We aimed to investigate not just whether the lower cognitive test scores of certain groups of individuals were associated with the GPS itself, but also whether the rate of cognitive decline was influenced by the joint interaction effect between time and GPSs, which are themselves differentiated over time.

## 2. Materials and Methods

### 2.1. Data

The WLS is based on 10,317 individuals surveyed in 1957—representing a 1/3 random sample of Wisconsin high school graduates that year—with randomly-selected siblings empaneled later. The study has collected 27,000+ phenotypic variables of the participants, ranging from cognition, personality, financial, and socioeconomic to genotype data during 6 waves of data collection over 60 years. The cohort represents non-Hispanic White Americans who completed at least 12 years of high school education in the United States. The participants underwent in-person, telephone structured interviews or mail-in questionnaires for each survey round after providing informed consent. All the instruments and operations were approved by the Institutional Review Board of the University of Wisconsin-Madison.

### 2.2. Genotype Data and Quality Control Process

From 2007–2008, saliva samples were collected by mail or during a home interview, and 9019 individuals were successfully genotyped at the Johns Hopkins University Center for Inherited Disease Research (CIDR) using the Illumina HumanOmniExpress-24 v.1.1 array designed for human genome build 37/hg19. The subsequent quality control process filtered individuals with (i) genotype missingness rate > 0.05 in all chromosomes, (ii) mismatch between recorded sex and genetically determined sex, (iii) high genetic relatedness with other individuals (>0.025), (iv) outlier in heterozygosity/homozygosity test, and (v) non-European ancestry outliers. Non-European individuals were identified by visually inspecting the principal component analysis (PCA) plot of the covariance matrix of the WLS genotype data with 1000 Genomes reference populations [32]. Additionally, SNPs with (i) genotype call rate < 0.95, (ii) Hardy-Weinberg exact test *p*-value < 1.0 × 10^−5^, and (iii) minor allele frequency < 0.01 were excluded from the data, resulting in 607,469 autosomal SNPs in 8527 European-ancestry individuals considered for further analysis. The data was then imputed to the Haplotype Reference Consortium (HRC) v1.1 European reference panel [32] and resulted in 39,127,657 variants. The detailed imputation and QC report are available separately [33,34].

### 2.3. Construction of Cognitive Capacity GPS

A set of cognitive ability-related GPSs were constructed based on four large-scale GWAS MTAG summary statistics on *educational attainment* (EA, *n* = 1,131,881), cognitive performance (CP, *n* = 257,841), self-reported math ability (MA, *n* = 564,698), and highest-level math class taken (HM, *n* = 430,445) from Lee et al. [18] and available from the WLS website upon request [30]. We downloaded the set of GPSs that was calculated with PLINK 1.9 [35] using the SNP weights adjusted for linkage disequilibrium using *LDpred* software [36]. All the SNP weights were obtained from cognitive GWAS discovery samples that did not contain the WLS participants.

### 2.4. Outcome Measures

#### 2.4.1. Cognitive Phenotypes

The participants’ cognition was assessed longitudinally using various tasks and structured questionnaires throughout the survey period of 60+ years. Our analysis used the participants’ cognition data from the four WLS survey rounds (taken in 1957, 1992–1994, 2003–2003, and 2011). The WLS data included the *IQ scores* of the participants from the Henmon-Nelson Test of Mental Ability with 90 items collected in their high school junior years in 1957, which measured general verbal, quantitative, and spatial knowledge [37,38,39], and their *high school class rank* percentile, which was based on the mean grade taken throughout the high school courses. The years of education (*educational attainment*) were calculated from the highest educational degree held by each participant at their middle age. We also included the cognition component of the Health Utilities Index 3 (*HUI3 cognition level*) which asked the subjects about their self-perceived cognitive status at the time of the interview.

Beginning in 1992–1994, 10 types of cognitive tasks were systematically proposed to the subjects at three time points over an 18–19 year period, including *similarities* (administered at survey timepoint 1/2/3), *letter fluency* (timepoint 2/3), *category fluency* (timepoint 2/3), *immediate recall* (timepoint 2/3), *delayed recall* (timepoint 2/3), *digit ordering* (timepoint 2/3), *number series* (timepoint 3), *linguistic function* (timepoint 3), including two health literacy assessments, the *Newest Vital Sign (NVS) Health Literacy Assessment* (timepoint 3) and the *Short Test of Functional Health Literacy in Adults (STOFHLA)* (timepoint 3). The phenotypes selected for the phenome-wide analysis are denoted with *italics* throughout the manuscript and their measurement criteria are available in the Supplementary Material. All the raw scores were z-scored for the analysis.

#### 2.4.2. Behavioral Phenotypes

The participants’ personality traits were assessed with the Big 5 Factor Model of Personality inventory test [40] in the WLS 1992–1994 collection wave. The five personality traits are known as one of the most common and influential models in the field of personality research and remain relatively stable over a lifetime. The Big 5 Factor Model of Personality test describes an individual’s personality in five basic dimensions: *extraversion, openness, neuroticism, conscientiousness, and agreeableness*. A higher score on each scale indicates the person has higher tendencies and behaviors representing the personality traits.

The subjects were asked to report on the time they spent participating in different leisure activities in hours per week or year. We compared various types of leisure activities including *reading, writing letters, watching movies/TV, light or vigorous physical activity (alone or together), doing crafts, hunting/fishing, playing a crossword puzzle/other word game*, *attending cultural events*, etc. The description of each leisure activity is provided in the Supplementary Material. To correct for outliers with extreme hours of certain activities, we took the natural logarithm of the reported hours for each activity and used it for the analysis.

In addition, we included two occupational standing variables collected in the WLS 2003–2005 wave based on their current or past employment information. The *occupational education score* was a numeric value of the types of industry or class-of-worker categories based on the 1990 US Census data, which indicated a percentage of persons who had at least a year of college education, ranging from 0 to 999. The *occupational income score* was calculated from the 1990-basis occupational earning scores, representing the percentage of persons in the 1990 US Census data in an industry or class-of-work category who earned more than $14.30/h in 1989, ranging from 37 to 876.

Since the IQ data of the participants’ spouses were available, we also included this variable for the analysis, hypothesizing that the behavior of assortative mating is associated with the GPSs of the cognitive abilities. Previous literature suggests the psychiatric hypothesis of assortative mating in academic achievements and IQ [41,42,43,44,45].

### 2.5. Statistical Analysis

#### 2.5.1. Cognitive/Behavioral PheWAS

Linear regression was used to investigate the associations between the four types of cognitive capacity GPSs (EA, CP, HM, and MA) and the normalized variables of the cognitive and behavioral phenotypes. Each cognitive capacity GPS was tested in separate models. We adjusted for biological sex, age, and the first 10 PCs of genetic ancestry and estimated each GPS’ significance (*p*-value), effect size (β), 95% confidence interval (CI), and proportion of variance explained (R^2^) for the target outcomes. Bonferroni-adjusted significance level of 2.60 × 10^−4^ was used to correct for multiple testing (48 tested phenotypes * 4 cognitive capacity GPS).

#### 2.5.2. Cognitive Changes

We selected 7 repetitive measures administered to the participants among aforementioned cognitive assessments, with an average interval of 6.5 years. We investigated its interaction effects with the cognitive capacity GPSs as the participants aged, including *similarities*, *letter fluency*, *category fluency*, *immediate recall*, *delayed recall*, *digit ordering,* and *HUI3 cognition level* (timepoint 2/3). Linear mixed-effects regressions were nested by the individual ID and each survey round (random effect) and we included the following fixed covariates in the analysis: age at the survey time point, biological sex, the first 10 ancestrally-informative principal components (PC1-10) of the genotype data and years of education. Bonferroni’s correction was used to adjust for multiple testing, and the scores were normalized except for the ordinal variable, *HUI3 cognition level*. We hypothesized that the contribution of genetic factors to the cognitive phenotypes was associated with the different degrees of cognitive decline in a particular cognitive domain. The analyses were performed in the R 3.5.1 environment, and the linear mixed-effect model was run with *lme4* package [46]. We calculated Schielzeth and Nakagawa’s R^2^ for generalized linear mixed effect models using r.squaredGLMM function from *MuMIn* R package [47,48,49].

## 3. Results

### 3.1. Participant Demographics

Our study included 8511 European-ancestry individuals with DNA genotype data, behavioral questionnaire data and cognitive assessment data available, including seven different cognitive ability tasks administered repetitively with an average interval of 6.5 years (SD = 1.25 year). The average age of the study participants was 48.6 at the time of the first round of the cognitive assessment (WLS survey round 4 (survey timepoint 1), 1992–1994, SD = 15.4 years), 64.2 at the second assessment (WLS survey round 5 (survey timepoint 2), 2003–2005, SD = 4.1 years), and 70.7 at the time of the last assessment (WLS survey round 6 (survey timepoint 3), 2011, SD = 4.2 years). The sample was 51.8% female, 47.8% completed high school or less than one year of college (number of years of education), and 78.2% were born in Wisconsin, USA.

### 3.2. PheWAS of Cognitive GPSs in the Cognitive/Behavioral Phenome

#### 3.2.1. Cognitive Phenotypes

**Figure 1:**
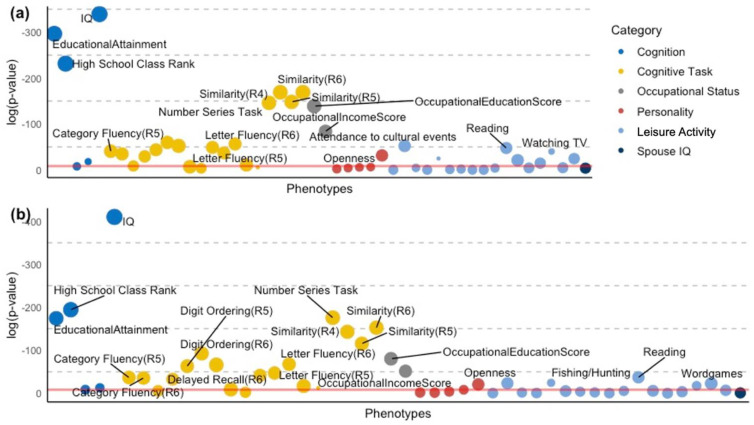
PheWAS plots of the *Educational attainment* (EA) and Cognitive Performance (CP) GPS in the Cognitive/behavioral phenome of the WLS participants. (**a**) PheWAS plot of *Educational Attainment* (EA) GPS. (**b**) PheWAS plot of Cognitive Performance (CP) GPS. The cognitive/behavioral phenotypes are presented on the x-axis. The phenotype variables were retrieved from the WLS survey data, primarily from the cognition and leisure activity modules in the 1957, 1992–1994, 2003–2005, and 2011 waves. The red line represents the phenome-wide significance level, og10 of the Bonferroni corrected *p*-value for multiple testing corrections (α = 0.05/(48 tested phenotypes * 4 GPS) = 2.60 × 10^−4^). The size of each point is proportional to the effect size of each cognitive capacity GPS-phenotype association.

Across all of the PheWAS results, IQ score showed the strongest association with the four cognitive GPSs in terms of the *p*-value and the increased proportion of variance explained (strongest with CP GPS, *p*-value = 7.74 × 10^−179^, β = 1.42, 95% CI = (1.32, 1.51)) (Figure 1, Table 1, Supplementary Table S1). The variance of the *IQ scores* explained by the CP GPS was 10.4% (Adjusted R^2^), whereas the baseline covariate model without the GPS variable explained 0.8% of the IQ score variance.

The years of *educational attainment* measure (strongest with EA GPS, *p*-value = 1.62 × 10^−129^, β = 1.73, 95% CI = (1.59, 1.87)) and the *high school class rank* (strongest with EA GPS, *p*-value = 3.07 × 10^−101^, β = 1.86, 95% CI = (1.69, 2.02)) also significantly associated with all four cognitive GPSs, following the IQ score. The variance of *high school class rank* explained by the EA GPS was 17.1% (Adjusted R^2^), whereas the baseline covariate model without the GPS variable explained 9.2% of the *high school class rank* variance.

Among the cognitive tasks, the *similarities* task presented the strongest statistical significance and positive effect size with the cognitive GPSs in all three rounds (strongest with timepoint3 *similarities* and EA GPS, *p*-value = 3.59 × 10^−74^, β = 1.36, 95% CI = (1.22, 1.51)). The cognitive GPSs also showed robust associations with the *number series* (strongest with HM GPS, *p*-value = 2.55 × 10^−78^, β = 0.94, 95% CI = (0.85, 1.04)) and digit ordering tasks (strongest with CP GPS, *p*-value = 8.63 × 10^−41^, β = 0.78, 95% CI = (0.67, 0.89)) across the different cognitive GPSs. Several cognitive tasks were also consistently and significantly associated across the cognitive GPSs with positive effect sizes, including *letter fluency* (strongest association with timpoint3 letter fluency and CP GPS, *p*-value = 5.01 × 10^−30^, β = 0.61, 95% CI = (0.50, 0.71)), *category fluency* (strongest association with timpoint3 category fluency and EA GPS, *p*-value = 2.03 × 10^−18^, β = 0.95, 95% CI = (0.74, 1.16)), *immediate recall* (strongest association with timpoint3 immediate recall and EA GPS, *p*-value = 5.29 × 10^−22^, β = 0.80, 95% CI = (0.64, 0.96)), *delayed recall* (strongest association with timpoint3 delayed recall and CP GPS, *p*-value = 5.00 × 10^−15^, β = 0.45, 95% CI = (0.34, 0.56)), *NVS Health Literacy assessments* (strongest with CP GPS, *p*-value = 2.28 × 10^−29^, β = 0.92, 95% CI = (0.76, 1.07)), which indicated that genetic contribution to cognitive abilities was positively correlated with higher cognitive scores for several assessments (Table 1).

#### 3.2.2. Behavioral Phenotypes

Among the Big 5 Personality traits, all the four GPS associations of openness (strongest with EA GPS, *p*-value = 2.19 × 10^−14^, β = 0.57, 95% CI = (0.42, 0.71)) met phenome-wide significance with positive effect sizes. In addition to openness, the HM and MA GPSs presented significant associations with neuroticism (strongest with MA GPS, *p*-value = 1.92 × 10^−6^, β = −0.32, 95% CI = (−0.45, −0.19)), showing negative effect sizes.

Leisure activities, such as reading books, magazines, newspapers or other reading material (strongest with EA GPS, *p*-value = 2.03 × 10^−21^, β = 0.50, 95% CI = (0.40, 0.60)) and attending cultural events (strongest with EA GPS, *p*-value = 2.06 × 10^−23^, β = 0.60, 95% CI = (0.49, 0.72)) presented phenome-wide significant associations across the cognitive GPSs with positive directions. Notably, watching TV (strongest with EA GPS, *p*-value = 4.16 × 10^−18^, β = −0.48, 95% CI = (−0.59, −0.37)) and fishing/hunting (strongest with EA GPS, *p*-value = 1.72 × 10^−11^, β = −0.59, 95% CI = (−0.77, −0.42)) showed significant negative associations with all the cognitive GPSs. Other phenome-wide significant activities included writing letters (strongest with EA GPS, *p*-value = 2.28 × 10^−11^, β = 0.32, 95% CI = (0.23, 0.41)), working on crosswords or word games (strongest with CP GPS, *p*-value = 1.27 × 10^−10^, β = 0.39, 95% CI = (0.27, 0.51)), and vigorous physical activities (alone) (strongest with EA GPS, *p*-value = 7.39 × 10^−10^, β = 0.61, 95% CI = (0.42, 0.80)) (Table 1, Figure 1).

Occupational education scores (strongest with EA GPS, *p*-value = 4.77 × 10^−61^, β = 1.22, 95% CI = (1.08, 0.36)) and occupational income scores (strongest with EA GPS, *p*-value = 3.50 × 10^−37^, β = 0.90, 95% CI = (0.76, 1.03)) presented positive relationships across all the cognitive GPSs. The association of the spouse’s IQ did not reach phenome-wide significance with any of the cognitive GPSs. The full PheWAS results of the phenome-wide significant associations are available in Supplementary Table S1.

### 3.3. Cognitive GPSs Correlate with Immediate Recall Changes

Our linear mixed effect model identified a significant age-x-GPS interaction effect on the *immediate recall* task. All four cognitive capacity GPSs showed significant interactions with the participants’ age (Age: GPS) for the *immediate recall* test scores (strongest with EA GPS, *p*-value = 1.79 × 10^−3^, β = 1.86 × 10^−1^) (Table 2). Their positive effect sizes suggested that an individual with a higher EA GPS tended to show fewer changes in the cognitive assessments as the individual aged.

**Figure 2:**
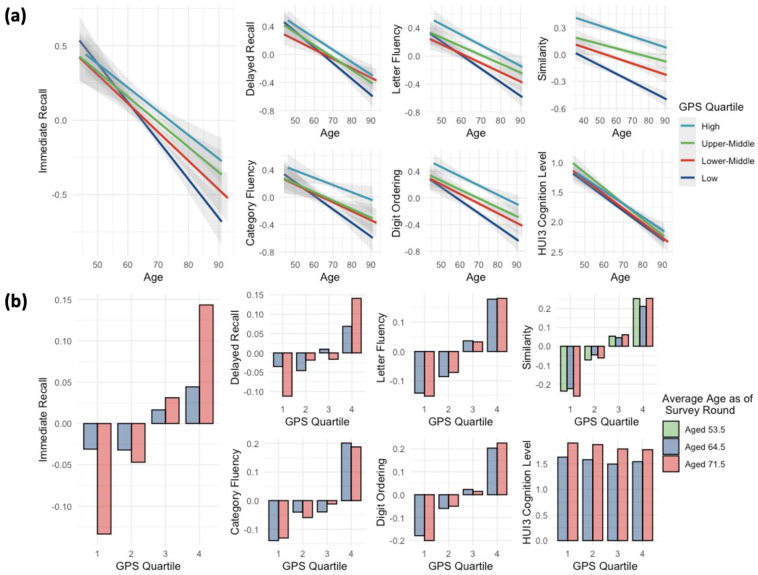
Graphical results of the linear mixed-effect model analysis showing that individuals with a higher cognitive GPS presented a slower trajectory of memory decline than those with a lower GPS. Changes in seven cognitive assessments (immediate recall task, category fluency task, digit ordering task, delayed recall task, letter fluency task, similarities task, and health utility index (HUI) level 3 cognition level) and the interaction effects of the CP GPS are shown. The selected seven cognitive assessments were repeatedly administered to 8511 European ancestry individuals between the average age of mid−50s (survey timepoint 1) and mid−70s (survey timepoint 3). (**a**) Interaction plots showing the different slopes of age−dependent interaction effects by the cognitive capacity GPS on the cognitive assessments. The x−axis indicates the age of the WLS participants at the survey timepoint, while the y-axis indicates each cognitive assessment score (z−scored). The four lines indicate the different slopes of the individuals’ cognitive changes stratified by GPS. The gray area represents the 95% confidence interval of each slope. The similarities task was the only task that was repeatedly administered to the participants since timepoint 1 (Average participants’ age 48.6). (**b**). Bar plots showing the stratification performance of the cognitive capacity GPS in each cognitive assessment module. Quartile 1 on the x-axis includes the individuals with the lowest cognitive GPS (bottom 25%) and Quartile 4 includes the individuals with the highest. The number on the y-axis represented the average phenotypic scores by each GPS quartile.

Compared to the individuals in the lowest GPS quartile, individuals in the highest GPS quartile showed a smaller decrease in their immediate recall score changes in later survey rounds. The slope of participants’ age in the lowest GPS quartile (β= −1.97 × 10^−1^, 95% CI = (−0.231, −0.163), *p*-value of slope = 8.61 × 10^−31^) distinctively showed a more intense decrease compared to the highest GPS quartile group (β = −1.24 × 10^−1^, 95% CI = (−0.158, −0.090), *p*-value of slope = 6.67 × 10^−13^) (Figure 2a). The pseudo-R^2^ of our linear mixed models, explaining the *immediate recall* by the cognitive capacity GPS, was up to 0.063 with fixed effects and was 0.170 with both fixed and random effects (both with EA GPS).

To visually depict the degree of cognitive changes according to GPS, we divided the cohort into four quartiles based on the GPS of each individual and analyzed the average phenotypic changes of each group over time. The average *immediate recall* task scores of the individuals in the highest GPS quartile were 0.044 (z-score) at timepoint 2 (average age of participants 64.5), and this increased to 0.073 (z-score) at timepoint 3 (average age 71.5) (1.65-fold increase). In contrast, the average task scores of the individuals in the lowest GPS quartile were −0.031 (z-score) at timepoint 2 and decreased to −0.077 (z-score) at timepoint 3 (2.48-fold decrease) (Figure 2b).

## 4. Discussion

In this study, we assessed the genetic influence of general cognitive abilities on cognitive and behavioral phenome using an integrative approach of GPS-based PheWAS on longitudinal observations in the aging population. We hypothesized that the contribution of genetic factors to cognitive capacities is associated with specific cognitive or behavioral phenotypes, and even different degrees of cognitive decline in certain cognitive domains.

Our study identified that the effects of the age-x-GPS interactions were significantly positive across all four cognitive capacity GPSs (Table 2), and individuals with a higher cognitive GPS had a slower trajectory of memory decline than those with a lower GPS (Figure 2a). This result indicates that the portion of the cognitive ability under genetic influence may serve as a ‘buffer’ against memory decline in aging. These observations align well with existing studies on the protective effect of education and intelligence on the occurrence of dementia [50]. A close relationship between early-life education and intelligence with cognitive decline has been reported for dementia and Alzheimer’s disease (AD) [51]. Even though it is not yet clear how early-life education and intelligence moderate the risk for dementia, our findings suggest that individual variations of memory decline are closely associated with the polygenic influences of cognitive abilities.

Among the repeated assessments of the seven cognitive domains with an average interval of 6.5 years, a decline in *immediate memory recall* during aging significantly correlated with the cognitive GPS. Memory recall, assessed by the *immediate* and *delayed recall* tests of words, is hippocampus-dependent [52,53,54]. We did not observe a significant interaction effect in the domain of *delayed recall*. It is interesting that the discovered genetic protective effect exerted specifically on the hippocampus-related immediate memory recall. There are two implications worth noting. Firstly, given the specificity of the correlations among the various cognitive domains, the genetic protective factor of immediate memory decline may be mediated via the hippocampus. Indeed, the hippocampus is the primary mediator of interventions for cognitive wellness or dementia, such as aerobic fitness [55], diet [56], and medication [57,58,59]. This is closely related to the unique role of the hippocampus in neurogenesis and synaptic plasticity [60,61]. Future research should thus test whether the hippocampus and hippocampal network underlies the genetic projective effect on immediate memory decline, but not in *delayed recall*, and if so seek to elucidate the mechanisms involved. Secondly, given the role of hippocampal memory impairment in the pathophysiology of AD, our finding may lead to a potential link of the inherited genetic factor of cognitive resilience to the individual differences in hippocampal degeneration, as well as memory decline in AD [4,62]. Testing this link will allow better stratification of AD and monitor the disease’s course by the individual-specific genetic profiles of cognitive resilience.

Our PheWAS identified several phenome-wide associations between cognitive capacity GPSs and cognitive assessments. The *similarities* task from WAIS, *number series* task, and *digit ordering* task showed the strongest associations across the four cognitive capacity GPSs regarding effect size (β) and *p*-value (Figure 1, Figure S1). These findings suggest that the cognitive components required to successfully complete the *similarities*, *number series*, or *digit ordering* tasks might strongly overlap with the genetic components of cognitive capacities primarily exhibited by the domain of fluid intelligence. The series of cognitive components involved in the *similarities* and *Number series* tasks, such as logical memory, symbol search, and reasoning, might be closely linked to early-life cognition, all of which may serve as phenotypic indicators for fluid intelligence. Our findings are backed up by the previous knowledge that fluid intelligence is considered to be more dependent on biological influences and less dependent on past learning experiences than crystallized intelligence [63].

Our analysis identified several phenome-wide significant associations of cognitive capacity GPSs with several early-life cognitive phenotypes, including *IQ scores, educational attainment,* or *high school class rank*. The significant genetic association between cognitive capacity and *IQ scores* or *educational attainment* has been well established in several GWAS studies on human intelligence [18,22,23,24,25]. The *IQ scores* of the WLS respondents were derived from the Henmon-Nelson test of mental ability, which is regarded as a general measure of overall intelligence, capturing both fluid and crystallized intelligence.

PheWAS of the behavioral phenome identified several behavioral traits highly related to genetic factors of cognitive capacity. All of the tested cognitive capacity GPSs positively correlated with *openness* among the Big 5 Personality factors, and some GPSs negatively correlated with *neuroticism* (Supplementary Table S1). The finding presents an interesting cross-trait hypothesis in which variances in personality dimensions may be partially explained by the genomic components of cognitive capacity or vice versa. ‘*Openness*’ could be regarded as the attitude and tendency to explore, detect, understand, and appreciate complicated new information patterns through both the senses and in the abstract [64]. Previous studies support our findings, concluding that an overall open-minded attitude might positively influence the long-term variances of cognitive abilities with the willingness to explore [65]. Not only for the Big 5 Personality factors, but overall, we believe that our PheWAS findings could be developed further for examining several cross-trait hypotheses related to human cognition in future studies.

No significant associations between *spouse IQ* and cognitive abilities were identified, which indicates that the behavioral associations between assortative mating and cognitive abilities are unclear. In addition, a strong relationship between occupational income and several cognitive capacity GPSs was found, which supports existing studies demonstrating a strong association between general mental ability and job performance [66].

A few limitations of this study should be noted. The WLS included two time points for measuring changes in their cognitive assessments with an average interval of 6.5 years. Adding more cognitive measurements through time will strengthen our findings by more thoroughly monitoring cognitive changes over a lifetime. Also, the unexplored impact of other sociodemographic variables such as socioeconomic status, educational environment, lifestyles, or family structure, should be considered to better connect our theoretical findings with the phenome-wide expression of cognitive abilities. In addition, we used European-ancestry-specific summary statistics to construct the cognitive capacity GPSs and applied them to the participants of European ancestry. Researchers the should note that application of our findings to non-European populations could be different, thus the results should be interpreted with caution. Future investigation is needed to elucidate the generalizability of our findings across diverse ancestry groups. Lastly, shared variance among the cognitive and behavioral phenome may interrupt the discovered genotype-phenotype associations. However, our correlation analysis (Supplementary Figure S2), revealed only a few extreme correlations (r > 0.5) among the distinct phenotypes except when comparing measurements to different instances of the same test. The results should be interpreted with caution considering the shared variances of tested phenotypes. Our findings could serve as the first cognitive-phenome map that describes the functional boundaries and behavioral implications of human cognition from a genetic perspective, and the map could be further expanded with the advanced phenotyping of human cognition and behavior traits.

## Figures and Tables

**Table 1:** Phenome-wide association studies (PheWAS) analysis for the four cognitive capacity GPSs with the cognitive/behavioral phenotypes. The table presents only the phenotypes significantly associated with all four cognitive capacity GPSs (*Educational attainment* (EA), Cognitive Performance (CP), Math Ability (MA), and Highest Math Class (HM)). The top cognitive capacity GPS-phenotype associations are presented from the full PheWAS results (available in Supplementary Table S1). Positive β (effect size) indicates that the genetic contribution to cognitive capacities is positively correlated with a higher score for each measurement module. The significance level of *p* < 2.60 × 10^−4^ was used according to the Bonferroni correction.

	Category	Strongest Cognitive Capacity GPS	β	95% CI* (lower)	95% CI* (upper)	*p*	Adjusted R^2^
*IQ*	Cognition	CP GPS	1.42	1.32	1.51	7.74 × 10^−179^	10.4%
*High School Class Rank*	Cognition	EA GPS	1.86	1.69	2.02	3.07 × 10^−101^	17.1%
*EducationalAttainment*	Cognition	EA GPS	1.73	1.59	1.87	1.62 × 10^−129^	12.0%
HUI3 Cognition Level (R6)**	Cognition	HM GPS	−0.32	−0.42	−0.22	2.63 × 10^−10^	1.2%
*Immediate Recall (R6)*	Cognitive Task	EA GPS	0.80	0.64	0.96	5.29 × 10^−22^	8.6%
*Similarity (R4)*	Cognitive Task	EA GPS	1.35	1.20	1.49	3.86 × 10^−74^	5.1%
*Similarity (R5)*	Cognitive Task	EA GPS	1.27	1.12	1.41	5.05 × 10^−65^	5.6%
*Similarity (R6)*	Cognitive Task	EA GPS	1.36	1.22	1.51	3.59 × 10^−74^	6.1%
*Number Series Task*	Cognitive Task	HM GPS	0.94	0.85	1.04	2.55 × 10^−78^	7.9%
*Category Fluency (R5)*	Cognitive Task	EA GPS	0.95	0.74	1.16	2.03 × 10^−18^	4.9%
*Category Fluency (R6)*	Cognitive Task	CP GPS	0.61	0.46	0.75	3.45 × 10^−16^	6.0%
*Delayed Recall (R6)*	Cognitive Task	CP GPS	0.45	0.34	0.56	5.00 × 10^−15^	7.9%
*Digit Ordering (R5)*	Cognitive Task	CP GPS	0.65	0.53	0.76	4.51 × 10^−28^	3.3%
*Digit Ordering (R6)*	Cognitive Task	CP GPS	0.78	0.67	0.89	8.63 × 10^−41^	5.0%
*Health Literacy Task (NVS)*	Cognitive Task	CP GPS	0.92	0.76	1.07	2.28 × 10^−29^	10.0%
*Letter Fluency (R5)*	Cognitive Task	EA GPS	0.73	0.56	0.90	7.98 × 10^−17^	4.2%
*Letter Fluency (R6)*	Cognitive Task	CP GPS	0.61	0.50	0.71	5.01 × 10^−30^	5.5%
*OccupationalEducationScore*	Occupational Status	EA GPS	1.22	1.08	1.36	4.77 × 10^−61^	4.8%
*OccupationalIncomeScore*	Occupational Status	EA GPS	0.90	0.76	1.03	3.50 × 10^−37^	13.4%
*Watching TV*	Leisure Activity	EA GPS	−0.48	−0.59	−0.37	4.16 × 10^−18^	1.9%
*Reading*	Leisure Activity	EA GPS	0.50	0.40	0.60	2.03 × 10^−21^	4.8%
*Fishing/Hunting*	Leisure Activity	EA GPS	−0.59	−0.77	−0.42	1.72 × 10^−11^	15.3%
*Attendance to cultural events*	Leisure Activity	EA GPS	0.60	0.49	0.72	2.06 × 10^−23^	4.8%
*Openness*	Personality	EA GPS	0.57	0.42	0.71	2.19 × 10^−14^	4.0%

* CI = Confidence interval; ** Only for *HUI3 Cognition Level* module, lower score indicates better cognition level.

**Table 2:** Linear mixed-effects model analysis results for the temporal changes of the *immediate recall* assessment score of the WLS participants according to the cognitive capacity GPSs, including *Educational attainment* (EA), Cognitive Performance (CP), Math Ability (MA), and Highest Math Class (HM) with the age interaction effect. The effect size of each variable is presented with the 95% confidence interval in parentheses.

	Dependent Variable			
	Immediate Recall			
	Educational Attainment GPS	Cognitive Performance GPS	Math Ability GPS	Highest Math Class Taken GPS
**Age**	−0.108 ***	−0.161 ***	−0.160 ***	−0.161 ***
	(−0.149, −0.066)	(−0.185, −0.136)	(−0.185, −0.136)	(−0.185, −0.136)
**GPS**	0.204 ***	0.032 ***	0.021*	0.028 **
	(0.077, 0.331)	(0.013, 0.051)	(0.002, 0.040)	(0.009, 0.047)
**Years of Educational Attainment**	0.069 ***	0.070 ***	0.072***	0.070 ***
	(0.061, 0.077)	(0.062, 0.078)	(0.064, 0.079)	(0.062, 0.078)
**Sex**	0.148 ***	0.149 ***	0.150 ***	0.149 ***
	(0.111, 0.186)	(0.111, 0.186)	(0.112, 0.187)	(0.111, 0.187)
**Age:GPS interaction**	0.186 ***	0.027 **	0.026 **	0.035 ***
	(0.069, 0.303)	(0.009, 0.046)	(0.007, 0.044)	(0.017, 0.053)
**Log likelihood**	−15,805.540	−15,809.350	−15,812.970	−15,808.150
**Akaike Inf. Crit.**	31,649.08	31,656.71	31,663.94	31,654.31
**Bayesian Inf. Crit.**	31,788.53	31,796.16	31,803.39	31,793.75

Note: ** *p* < 0.01; *** *p* < 0.002, 0.002 = Bonferroni-adjusted Significance Level with 7 cognitive modules * 4 GPS tested.

## Data Availability

The data/analyses presented in the current publication have been deposited in and are available from the dbGaP database under dbGaP accession phs001157.v1.p1.

## Supplementary Materials

The following are available online at https://www.mdpi.com/article/10.3390/genes13081320/s1, Figure S1: Distribution of the cognitive/behavioral phenotypes; Figure S2: Correlation matrix of the cognitive/behavioral phenotypes and the cognitive capacity GPSs. Table S1: Phenome-wide Significant Results of Cognitive Capacity GPSs on the Cognitive/Behavioral Phenome (*p* < 2.60 × 10^−4^, Bonferroni corrected); File S1: Supplementary Method: survey Instruments for creating the Cognitive/Behavioral Phenome [37,38,39,40,41,42,43,44,45,67,68,69,70,71,72].

## References

[1] Deary I.J., Yang J., Davies G., Harris S.E., Tenesa A., Liewald D., Luciano M., Lopez L.M., Gow A.J., Corley J. (2012). Genetic contributions to stability and change in intelligence from childhood to old age. Nature.

[2] Sharp E.S., Gatz M. (2011). Relationship between education and dementia: An updated systematic review. Alzheimer Dis. Assoc. Disord..

[3] Stern Y. (2002). What is cognitive reserve? Theory and research application of the reserve concept. J. Int. Neuropsychol. Soc..

[4] Stern Y. (2012). Cognitive reserve in ageing and Alzheimer’s disease. Lancet Neurol..

[5] Stern Y. (2009). Cognitive reserve. Neuropsychologia.

[6] Melikyan Z.A., Corrada M.M., Leiby A.M., Sajjadi S.A., Bukhari S., Montine T.J., Kawas C.H. (2022). Cognitive resilience to three dementia-related neuropathologies in an oldest-old man: A case report from The 90+ Study. Neurobiol. Aging.

[7] Bolton A., Yaroush R., Staal M., Bourne L. (2008). Cognitive Performance and Resilience to Stress. Biobehavioral Resilience to Stress.

[8] Yu L., Petyuk V.A., Gaiteri C., Mostafavi S., Young-Pearse T., Shah R.C., Buchman A.S., Schneider J.A., Piehowski P.D., Sontag R.L. (2018). Targeted brain proteomics uncover multiple pathways to Alzheimer’s dementia. Ann. Neurol..

[9] Yu L., Tasaki S., Schneider J.A., Arfanakis K., Duong D.M., Wingo A.P., Wingo T.S., Kearns N., Thatcher G.R.J., Seyfried N.T. (2020). Cortical Proteins Associated With Cognitive Resilience in Community-Dwelling Older Persons. JAMA Psychiatry.

[10] Andrews S.J., Das D., Cherbuin N., Anstey K.J., Easteal S. (2016). Association of genetic risk factors with cognitive decline: The PATH through life project. Neurobiol. Aging.

[11] Morley J.F., Xie S.X., Hurtig H.I., Stern M.B., Colcher A., Horn S., Dahodwala N., Duda J.E., Weintraub D., Chen-Plotkin A.S. (2012). Genetic influences on cognitive decline in Parkinson’s disease. Mov. Disord..

[12] Raj T., Chibnik L.B., McCabe C., Wong A., Replogle J.M., Yu L., Gao S., Unverzagt F.W., Stranger B., Murrell J. (2017). Genetic architecture of age-related cognitive decline in African Americans. Neurol. Genet..

[13] Zammit A.R., Yu L., Petyuk V., Schneider J.A., De Jager P.L., Klein H.U., Bennett D.A., Buchman A.S. (2022). Cortical Proteins and Individual Differences in Cognitive Resilience in Older Adults. Neurology.

[14] Mostafavi S., Gaiteri C., Sullivan S.E., White C.C., Tasaki S., Xu J., Taga M., Klein H.-U., Patrick E., Komashko V. (2018). A molecular network of the aging human brain provides insights into the pathology and cognitive decline of Alzheimer’s disease. Nat. Neurosci..

[15] Furlong L.I. (2013). Human diseases through the lens of network biology. Trends Genet..

[16] Hindorff L.A., Sethupathy P., Junkins H.A., Ramos E.M., Mehta J.P., Collins F.S., Manolio T.A. (2009). Potential etiologic and functional implications of genome-wide association loci for human diseases and traits. Proc. Natl. Acad. Sci. USA.

[17] Chakravarti A., Turner T.N. (2016). Revealing rate-limiting steps in complex disease biology: The crucial importance of studying rare, extreme-phenotype families. Bioessays.

[18] Lee J.J., Wedow R., Okbay A., Kong E., Maghzian O., Zacher M., Nguyen-Viet T.A., Bowers P., Sidorenko J., Karlsson Linner R. (2018). Gene discovery and polygenic prediction from a genome-wide association study of educational attainment in 1.1 million individuals. Nat. Genet..

[19] Rietveld C.A., Medland S.E., Derringer J., Yang J., Esko T., Martin N.W., Westra H.J., Shakhbazov K., Abdellaoui A., Agrawal A. (2013). GWAS of 126,559 individuals identifies genetic variants associated with educational attainment. Science.

[20] Okbay A., Beauchamp J.P., Fontana M.A., Lee J.J., Pers T.H., Rietveld C.A., Turley P., Chen G.B., Emilsson V., Meddens S.F. (2016). Genome-wide association study identifies 74 loci associated with educational attainment. Nature.

[21] Plomin R., von Stumm S. (2018). The new genetics of intelligence. Nat. Rev. Genet..

[22] Trampush J.W., Yang M.L.Z., Yu J., Knowles E., Davies G., Liewald D.C., Starr J.M., Djurovic S., Melle I., Sundet K. (2017). GWAS meta-analysis reveals novel loci and genetic correlates for general cognitive function: A report from the COGENT consortium. Mol. Psychiatry.

[23] Sniekers S., Stringer S., Watanabe K., Jansen P.R., Coleman J.R.I., Krapohl E., Taskesen E., Hammerschlag A.R., Okbay A., Zabaneh D. (2017). Genome-wide association meta-analysis of 78,308 individuals identifies new loci and genes influencing human intelligence. Nat. Genet..

[24] Davies G., Marioni R.E., Liewald D.C., Hill W.D., Hagenaars S.P., Harris S.E., Ritchie S.J., Luciano M., Fawns-Ritchie C., Lyall D. (2016). Genome-wide association study of cognitive functions and educational attainment in UK Biobank (N = 112,151). Mol. Psychiatry.

[25] Davies G., Armstrong N., Bis J.C., Bressler J., Chouraki V., Giddaluru S., Hofer E., Ibrahim-Verbaas C.A., Kirin M., Lahti J. (2015). Genetic contributions to variation in general cognitive function: A meta-analysis of genome-wide association studies in the CHARGE consortium (N = 53,949). Mol. Psychiatry.

[26] Davies G., Tenesa A., Payton A., Yang J., Harris S.E., Liewald D., Ke X., Le Hellard S., Christoforou A., Luciano M. (2011). Genome-wide association studies establish that human intelligence is highly heritable and polygenic. Mol. Psychiatry.

[27] Bouchard T.J., McGue M. (2003). Genetic and environmental influences on human psychological differences. J. Neurobiol..

[28] Rietveld C.A., Esko T., Davies G., Pers T.H., Turley P., Benyamin B., Chabris C.F., Emilsson V., Johnson A.D., Lee J.J. (2014). Common genetic variants associated with cognitive performance identified using the proxy-phenotype method. Proc. Natl. Acad. Sci. USA.

[29] Trampush J.W., Lencz T., Knowles E., Davies G., Guha S., Pe’er I., Liewald D.C., Starr J.M., Djurovic S., Melle I. (2015). Independent evidence for an association between general cognitive ability and a genetic locus for educational attainment. Am. J. Med. Genet. B Neuropsychiatr. Genet..

[30] Herd P., Carr D., Roan C. (2014). Cohort profile: Wisconsin longitudinal study (WLS). Int. J. Epidemiol..

[31] Carney A.K. (2014). Wisconsin longitudinal study. Int. J. Aging Hum. Dev..

[32] McCarthy S., Das S., Kretzschmar W., Delaneau O., Wood A.R., Teumer A., Kang H.M., Fuchsberger C., Danecek P., Sharp K. (2016). A reference panel of 64,976 haplotypes for genotype imputation. Nat. Genet..

[33] Okbay A., Benjamin D., Visscher P. Documentation for the Data of Educational Attainment, Cognitive Performance and Math-Related Scores. https://www.ssc.wisc.edu/wlsresearch/documentation/GWAS/Lee_et_al_(2018)_PGS_WLS.pdf.

[34] Center T.U.o.W.G.A. (2016). A Longitudinal Resource for Genetic Research in Behavioral and Health Sciences—Imputation Report Wisconsin Longitudinal Study Nov 2, 2016. https://www.ssc.wisc.edu/wlsresearch/documentation/GWAS/Herd_1000G_IMPUTE2report.pdf.

[35] Purcell S., Neale B., Todd-Brown K., Thomas L., Ferreira M.A., Bender D., Maller J., Sklar P., de Bakker P.I., Daly M.J. (2007). PLINK: A tool set for whole-genome association and population-based linkage analyses. Am. J. Hum. Genet..

[36] Vilhjalmsson B.J., Yang J., Finucane H.K., Gusev A., Lindstrom S., Ripke S., Genovese G., Loh P.R., Bhatia G., Do R. (2015). Modeling Linkage Disequilibrium Increases Accuracy of Polygenic Risk Scores. Am. J. Hum. Genet..

[37] Hauser R.M., Palloni A. (2011). Adolescent IQ and Survival in the Wisconsin Longitudinal Study. J. Gerontol. Ser. B.

[38] Henmon V. (1946). Henmon-Nelson Tests of Mental Ability, High School Examination-Grades 7 to 12-Forms a, b, and c. Teacher’s Manual.

[39] Henmon V.A.C., Holt F.O. (1931). A Report on the Administration of Scholastic Aptitude Tests to 34,000 High. School Seniors in Wisconsin in 1929 and 1930: Prepared for the Committee on Cooperation, Wisconsin Secondary Schools and Colleges.

[40] John O.P., Donahue E.M., Kentle R.L. (1991). The Big Five Inventory—Versions 4a and 54.

[41] Halpin B. (2003). Educational homogamy in Ireland and Britain: Trends and patterns. Br. J. Sociol..

[42] Mascie-Taylor C.G., Vandenberg S.G. (1988). Assortative mating for IQ and personality due to propinquity and personal preference. Behav. Genet..

[43] Watson D., Klohnen E.C., Casillas A., Simms E.N., Haig J., Berry D.S. (2004). Match makers and deal breakers: Analyses of assortative mating in newlywed couples. J. Pers..

[44] Hur Y.M. (2003). Assortive mating for personaltiy traits, educational level, religious affiliation, height, weight, adn body mass index in parents of Korean twin sample. Twin Res..

[45] Pan Y., Wang K. (2011). Spousal concordance in academic achievements and IQ a principal component analysis. Open J. Psychiatry.

[46] Bates D., Mächler M., Bolker B., Walker S. (2015). Fitting Linear Mixed-Effects Models Using lme4. J. Stat. Softw..

[47] Johnson P.C.D. (2014). Extension of Nakagawa & Schielzeth’s R2GLMM to random slopes models. Methods Ecol. Evol..

[48] Nakagawa S., Johnson P.C., Schielzeth H. (2017). The coefficient of determination R 2 and intra-class correlation coefficient from generalized linear mixed-effects models revisited and expanded. J. R. Soc. Interface.

[49] Nakagawa S., Schielzeth H. (2013). A general and simple method for obtaining R2 from generalized linear mixed-effects models. Methods Ecol. Evol..

[50] Plassman B.L., Welsh K.A., Helms M., Brandt J., Page W.F., Breitner J.C. (1995). Intelligence and education as predictors of cognitive state in late life: A 50-year follow-up. Neurology.

[51] Katzman R. (1993). Education and the prevalence of dementia and Alzheimer’s disease. Neurology.

[52] Griffith H.R., Pyzalski R.W., O’Leary D., Magnotta V., Bell B., Dow C., Hermann B., Seidenberg M. (2003). A controlled quantitative MRI volumetric investigation of hippocampal contributions to immediate and delayed memory performance. J. Clin. Exp. Neuropsychol..

[53] Golomb J., Kluger A., de Leon M.J., Ferris S.H., Convit A., Mittelman M.S., Cohen J., Rusinek H., De Santi S., George A.E. (1994). Hippocampal formation size in normal human aging: A correlate of delayed secondary memory performance. Learn. Mem..

[54] Hackert V.H., den Heijer T., Oudkerk M., Koudstaal P.J., Hofman A., Breteler M.M. (2002). Hippocampal head size associated with verbal memory performance in nondemented elderly. Neuroimage.

[55] Erickson K.I., Prakash R.S., Voss M.W., Chaddock L., Hu L., Morris K.S., White S.M., Wojcicki T.R., McAuley E., Kramer A.F. (2009). Aerobic fitness is associated with hippocampal volume in elderly humans. Hippocampus.

[56] Latimer C.S., Brewer L.D., Searcy J.L., Chen K.C., Popovic J., Kraner S.D., Thibault O., Blalock E.M., Landfield P.W., Porter N.M. (2014). Vitamin D prevents cognitive decline and enhances hippocampal synaptic function in aging rats. Proc. Natl. Acad. Sci. USA.

[57] Goveas J.S., Xie C., Ward B.D., Wu Z., Li W., Franczak M., Jones J.L., Antuono P.G., Li S.J. (2011). Recovery of hippocampal network connectivity correlates with cognitive improvement in mild Alzheimer’s disease patients treated with donepezil assessed by resting-state fMRI. J. Magn. Reson. Imaging.

[58] Jin K., Peel A.L., Mao X.O., Xie L., Cottrell B.A., Henshall D.C., Greenberg D.A. (2004). Increased hippocampal neurogenesis in Alzheimer’s disease. Proc. Natl. Acad. Sci. USA.

[59] Krishnan K.R., Charles H.C., Doraiswamy P.M., Mintzer J., Weisler R., Yu X., Perdomo C., Ieni J.R., Rogers S. (2003). Randomized, placebo-controlled trial of the effects of donepezil on neuronal markers and hippocampal volumes in Alzheimer’s disease. Am. J. Psychiatry.

[60] Toda T., Gage F.H. (2018). Review: Adult neurogenesis contributes to hippocampal plasticity. Cell Tissue Res..

[61] Snyder J.S., Kee N., Wojtowicz J.M. (2001). Effects of adult neurogenesis on synaptic plasticity in the rat dentate gyrus. J. Neurophysiol..

[62] Scarmeas N., Albert S.M., Manly J.J., Stern Y. (2006). Education and rates of cognitive decline in incident Alzheimer’s disease. J. Neurol. Neurosurg. Psychiatry.

[63] Akshoomoff N., Beaumont J.L., Bauer P.J., Dikmen S.S., Gershon R.C., Mungas D., Slotkin J., Tulsky D., Weintraub S., Zelazo P.D. (2013). VIII. NIH Toolbox Cognition Battery (CB): Composite scores of crystallized, fluid, and overall cognition. Monogr. Soc. Res. Child Dev..

[64] DeYoung C.G. (2015). Openness/intellect: A dimension of personality reflecting cognitive exploration. APA Handbook of Personality and Social Psychology, Volume 4: Personality Processes and Individual Differences. APA Handbooks in Psychology^®^.

[65] DeYoung C.G., Peterson J.B., Higgins D.M. (2005). Sources of openness/intellect: Cognitive and neuropsychological correlates of the fifth factor of personality. J. Pers..

[66] Schmidt F.L., Hunter J. (2004). General mental ability in the world of work: Occupational attainment and job performance. J. Pers. Soc. Psychol..

[67] Wechsler D. (1981). The Wechsler Adult Intelligence Acale—Revised.

[68] Wechsler D. (1997). WAIS-III, Wechsler Adult Intelligence Scal. Administration and Scoring Manual.

[69] Hubbard R.C. Newest Vital Sign (NVS). https://pfe-pfizercom-prod.s3.amazonaws.com/health/nvs_flipbook_english_final.pdf.

[70] Maenner M.J., Greenberg J.S., Mailick M.R. (2015). Association Between Low IQ Scores and Early Mortality in Men and Women: Evidence from a Population-Based Cohort Study. Am. J. Intellect. Dev. Disabil..

[71] Henmon V.A.C., Nelson M.J. (1954). The Henmon-Nelson tests of mental ability. Manual for Administration.

[72] Willingham W.W., Pollack J.M., Lewis C. (2002). Grades and test scores: Accounting for observed differences. J. Educ. Meas..

