# Supplementary figures and images for: Cognitive Capacity Genome-Wide Polygenic Scores Identify Individuals with Slower Cognitive Decline in Aging
Source: Genes (Basel). 2022 Jul 24;13(8):1320. doi: 10.3390/genes13081320 (PMC9331374; doi:10.3390/genes13081320)

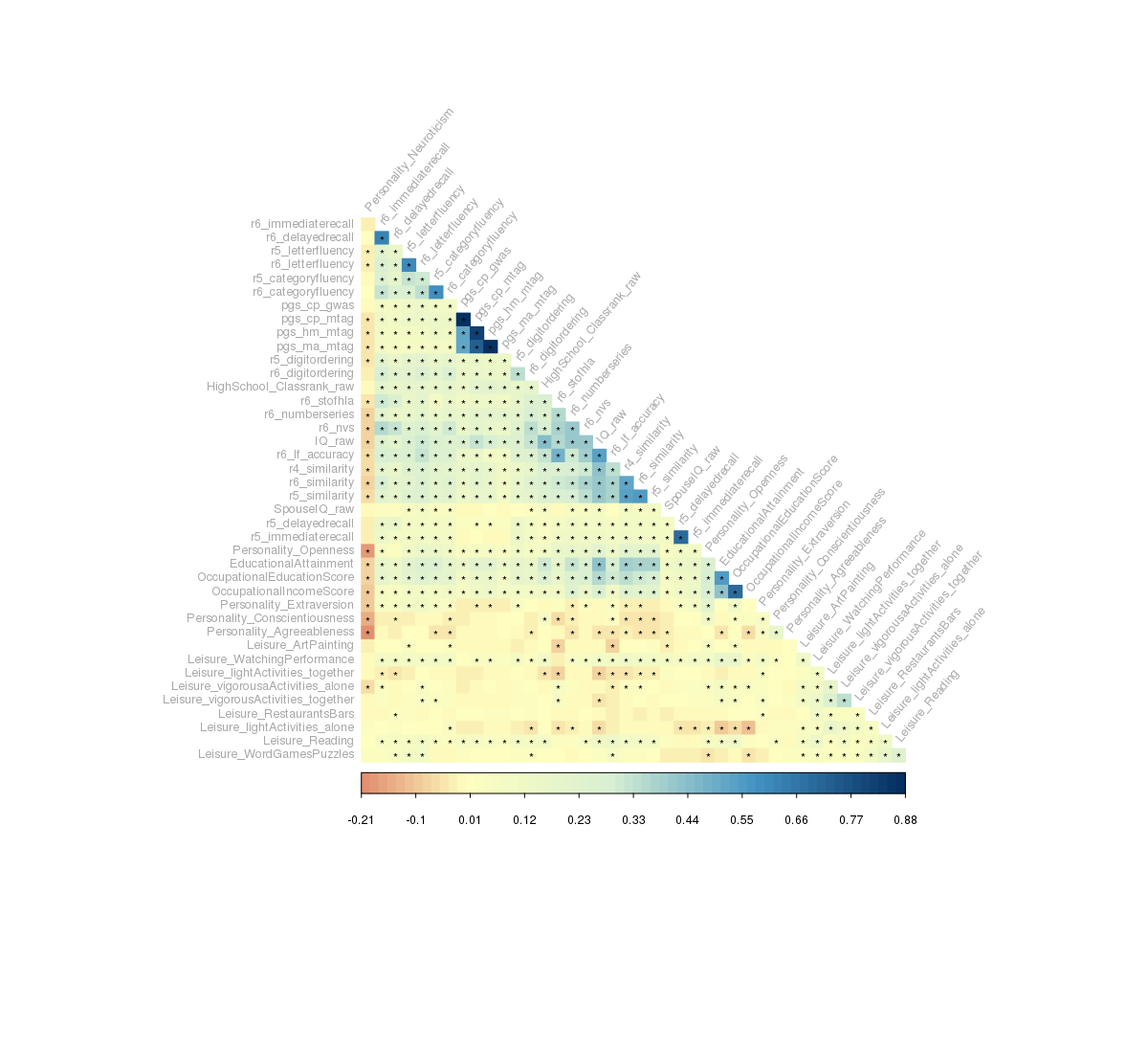

Supplement: Supplementary file 1 [file genes-13-01320-s001.zip › JooHayes_WLS_Supplement Figure S2_WLS_corrplot.png]
