# Supplementary material for: Cognitive Capacity Genome-Wide Polygenic Scores Identify Individuals with Slower Cognitive Decline in Aging
Source: Genes (Basel). 2022 Jul 24;13(8):1320. doi: 10.3390/genes13081320 (PMC9331374; doi:10.3390/genes13081320)
Supplement: Supplementary file 1 [file genes-13-01320-s001.zip › JooHayes_WLS_Supplementary Figure S1_Histogram_ComBio.zip!/JooHayes_WLS_Supplementary Figure S1_Histogram_ComBio.pdf]

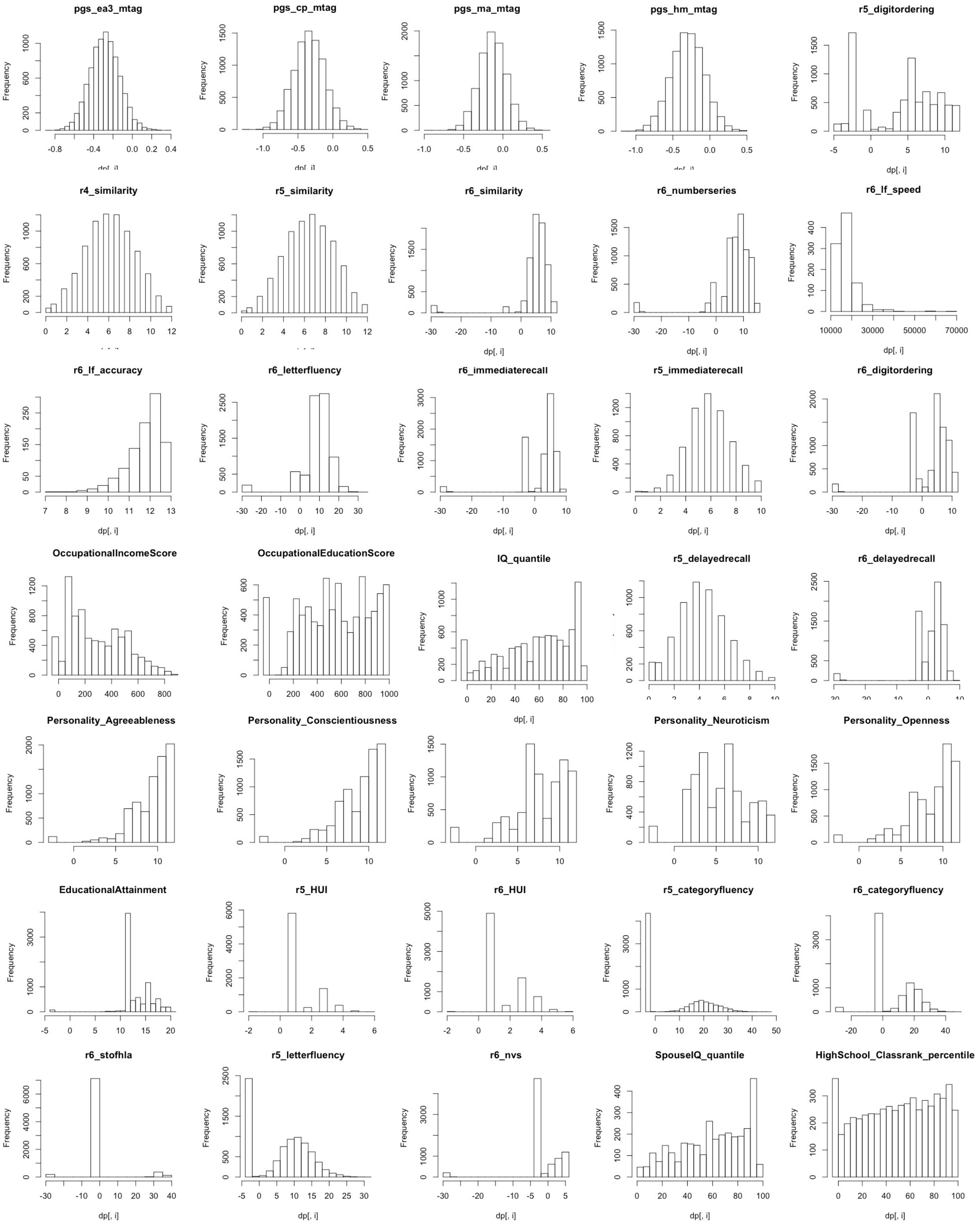
pgs_ea3_mtag
pgs_cp_mtag
pgs_ma_mtag
pgs_hm_mtag
r5_digitordering
r4_similarity
r5_similarity
r6_similarity
r6_numberseries
r6_lf_speed
r6_lf_accuracy
r6_letterfluency
r6_immediaterecall
r5_immediaterecall
r6_digitordering
OccupationalIncomeScore
OccupationalEducationScore
IQ_quantile
r5_delayedrecall
r6_delayedrecall
Personality_Agreeableness
Personality_Conscientiousness
Personality_Neuroticism
Personality_Openness
EducationalAttainment
r5_HUI
r6_HUI
r5_categoryfluency
r6_categoryfluency
r6_stofhla
r5_letterfluency
r6_nvs
SpouseIQ_quantile
HighSchool_Classrank_percentile
Frequency
dp[, i]

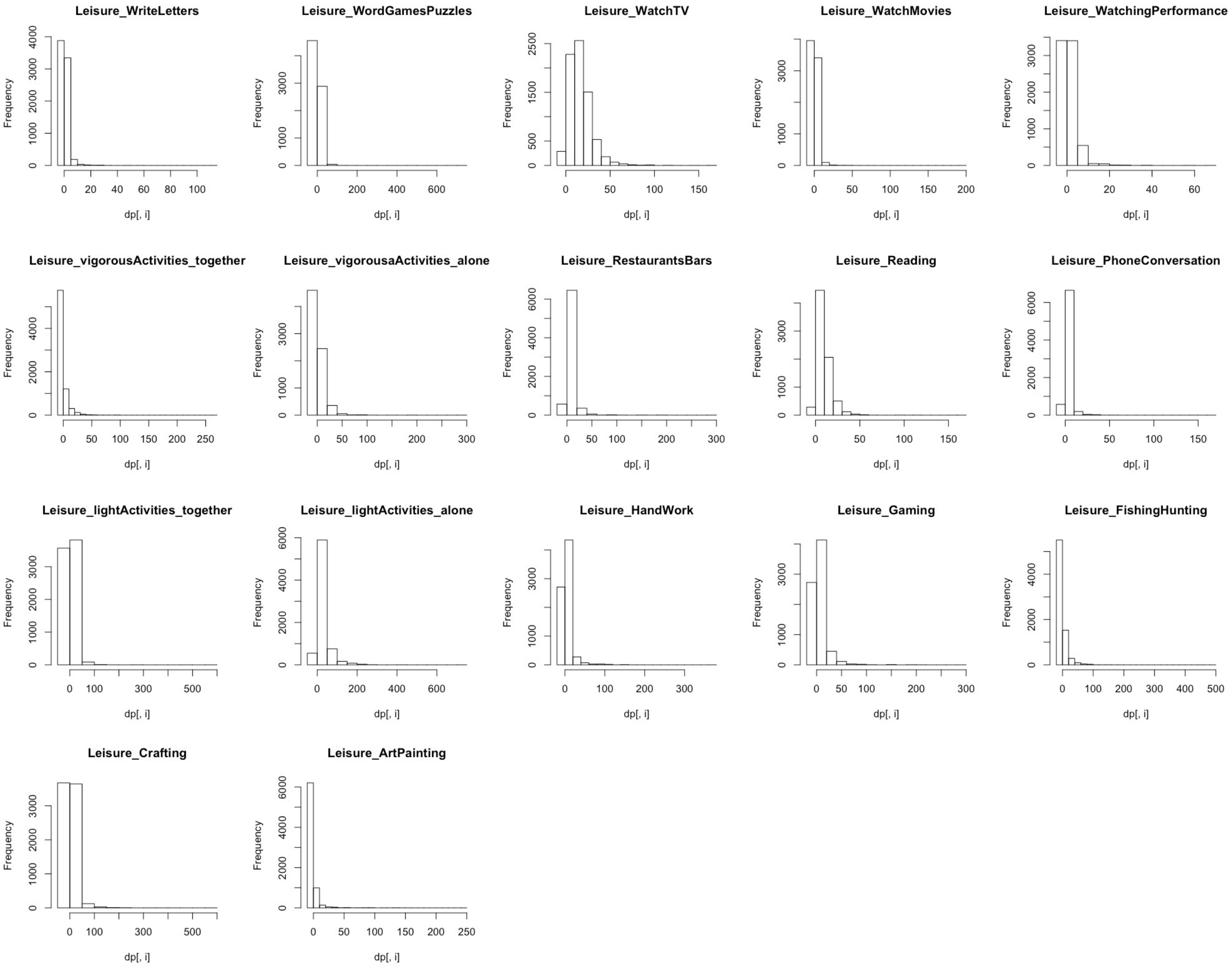
Leisure_WriteLetters
Leisure_WordGamesPuzzles
Leisure_WatchTV
Leisure_WatchMovies
Leisure_WatchingPerformance
Leisure_vigorousActivities_together
Leisure_vigorousaActivities_alone
Leisure_RestaurantsBars
Leisure_Reading
Leisure_PhoneConversation
Leisure_lightActivities_together
Leisure_lightActivities_alone
Leisure_HandWork
Leisure_Gaming
Leisure_FishingHunting
Leisure_Crafting
Leisure_ArtPainting
Frequency
dp[, i]
