# Supplementary material for: Cognitive Capacity Genome-Wide Polygenic Scores Identify Individuals with Slower Cognitive Decline in Aging
Source: Genes (Basel). 2022 Jul 24;13(8):1320. doi: 10.3390/genes13081320 (PMC9331374; doi:10.3390/genes13081320)
Supplement: Supplementary file 1 [file genes-13-01320-s001.zip › JooHayes_WLS_SupplementaryMaterial_ComBio_final.zip!/JooHayes_WLS_SupplementaryMaterial_ComBio_final.pdf]

Supplementary Material.

Cognitive Capacity Genome-wide Polygenic Scores Identify Individuals Resilient to Cognitive Decline in Aging

Running title: Polygenic underpinning of cognitive and behavioral phenotypes

Yoonjung Yoonie Joo, Ph.D, Jiook Cha, Ph.D, Jeremy Freese, Ph.D, M Geoffrey Hayes, Ph.D.

Supplementary Method. Description of each cognitive/behavioral phenotypes used for the cognitive capacity GPS-PheWAS.

Supplementary Table S1. Significant PheWAS results of EA (Educational attainment), CP (Cognitive Performance), HM (Highest Math), and MA (Math Ability) GPS in cognitive/behavioral phenome of the WLS participants.

Supplementary Figure S1. Distribution of each cognitive/behavioral phenotypes

File S1. Supplementary Method.

Survey Instruments for creating the Cognitive/Behavioral Phenome

Detailed information of each survey instrument can be searched and obtained in the official WLS website: https://www.ssc.wisc.edu/wlsresearch/documentation/

(1) **Cognitive Phenotypes –** Cognition

The cognition of the study participants was assessed longitudinally using various tasks and structured questionnaires throughout the survey period of 60+ years. Our analysis used the participants' cognition data from the four WLS survey rounds as early as 1957.

a. IQ score: All the WLS respondents completed the Henmon-Nelson Test of Mental Ability with 90 items during their high school junior years (1957), which included multiple-choice assessment for verbal, quantitative, and spatial knowledge [4,5]. It was part of mandatory assessment administered by all Wisconsin high schools and colleges from 1930s-1960s [6-8]. Raw scores were converted to IQ scores by standardizing to a mean of 100 based on Wisconsin centile rank (z-score).

b. Educational attainment: This metric from survey round 5 (2003-2005) calculates years of education estimated from the highest degree obtained by each participant. The measure ranges from 12 to 20 years, the lowest number indicates the education years of high school graduates and the highest one indicates the education years for PhD degree holders.

c. High School Class rank: Percentile rank information was retrieved from high school records, which is based on the mean grade taken throughout the high school courses. [100–(rank in class/(# of students in class))*100] The correlation between class grade and standardized test scores was as high as 0.90, which reduces the possibility of teacher bias on grades or ranks [9].

d. Health Utilities Index Mark 3 (*HUI3 cognition level*) which asked the subjects about their self-perceived cognitive status at the time of interview. Lower HUI3 cognition level score indicates better cognition level of individual.

a) 1 - Able to remember most things, think clearly and solve day to day problems.

b) 2 - Able to remember most things, but have a little difficulty when trying to think and solve day to day problems.
c) 3 - Somewhat forgetful, but able to think clearly and solve day to day problems.
d) 4 - Somewhat forgetful, and have a little difficulty when trying to think or solve day to day problems.
e) 5 - Very forgetful, and have great difficulty when trying to think or solve day to day problems.

(2) **Cognitive Phenotypes –** Cognitive assessments

Beginning in 1992-1994, the WLS administered 10 types of cognitive tasks to the participants, which were used for the creation of the cognitive phenome for our study: '*Similarities* (administered at survey timepoint 1/2/3), *Letter Fluency* (timepoint 2/3), *Category Fluency* (timepoint 2/3), *Immediate Recall* (timepoint 2/3), *Delayed Recall* (timepoint 2/3), *Digit Ordering* (timepoint 2,3), *Number Series* (timepoint 3), *Linguistic Function* (timepoint 3), including two health literacy assessment: *Newest Vital Sign (NVS) Health Literacy Assessment* (timepoint 3) and *Short Test of Functional Health Literacy in Adults (STOFHLA)* (timepoint 3). Usually, higher scores indicate a higher cognitive ability. Except for the one-time health literacy tests and *Number Series* tasks, the remaining 6 cognitive modules were repeatedly administered to the participants at average age 64.5 (2003-2005, WLS survey round 5, timepoint 2 in the main text) and age 71.5 (2011, WLS survey round 6, timepoint 3 in the main text).

a. *Similarities* (administered at survey timepoint 1/2/3): The Weschler Adult Intelligence Scale (WAIS) similarity test asked how two presented choices are alike, such as "in what way are an orange and a banana alike" [1]. The module was the only cognition test that was repeatedly administered to the WLS respondents three times throughout the survey period: timepoint1 (average age 48.6, 1992-1994), timepoint2 and timepoint3. A total of six tasks was assigned with scores of 0, 1, 2 based on the number of correct answers. In sum, the full similarity score ranged from 0 to 12.

b. *Letter Fluency* (timepoint 2/3), *Category Fluency* (timepoint 2/3): The linguistic fluency module asked participants to present all the words that begin with the letter 'F' or 'L' in the one minute (letter), and all the words that fall into the categories of 'animals' or 'fruits' in the one minute (category).

c. *Immediate Recall* (timepoint 2/3), *Delayed Recall* (timepoint 2/3): Word recall task consisted of two parts to test the respondent's memorizing ability; first, the interviewer read aloud a set of ten non-repetitive words and immediately asked the participants to recall as many words as they could remember from the original list (immediate word recall). Without notice, after 12 minutes of continuing another interview, the interviewer asked the participants to repeat back the words they could remember (delayed word recall). The number of correctly recalled words were recorded.

d. *Linguistic Function* (timepoint 3): The linguistic functioning modules were comprised of six cognitive and language tasks that measured the linguistic accuracy and latency of the respondents: rapid automated naming, word identification, picture naming, number judgement and reverse number judgment, word recognition and category verification. Rapid automated naming task required the respondents to sequentially align a given list of 36 digits as quickly as possible, measuring the processing speed of numerical objects. Word identification task asked the respondents to pronounce 10 regular words, 10 irregular pronounced words, and 10 nonsense words as quickly and precisely as possible. Picture naming task assessed verbal production skills by asking them to identify the human/animal objects in 24 pictures shown on a screen. Number judgment task measured the processing speed of numerical decision, by asking respondents to decide instantly if a shown digit on a screen is bigger or smaller than 5, for 16 different digits. Word recognition task presented 24 sets of words on the screen and asked participants to choose the real word in a timely manner. Category verification task showed 20 words followed by two pictures and asked the participants to indicate a similar picture matched to the word. In addition to individual measures of each task (accuracy and reaction time in milliseconds), we used the summary measures for all six tasks.

e. *Digit Ordering* (timepoint 2,3): The task assigned the participants with random single digit numbers and asked them to reorder the numbers from smallest to largest, following the test guideline of WAIS-III digit backward test [2].

f. *Number Series* (timepoint 3): The McArdle & Woodcock number series task assessed numerical and inference ability of the respondents with additional emphasis on quantitative reasoning. Each task consisted of a series of integers (e.g. 3, 5, 8, 12, __) and asked the participants to identify the correct number to complete the series. Each participant completed a total of six items, and the difficulty of the test were determined by the number of correct answers from the first three questions.

g. Health literacy test score

a) The Short Test of Functional Health Literacy in Adults (STOFHLA) (timepoint 3) module is a timed reading comprehension assessment for healthcare terminologies that is used in a conventional medical setting, such as a Medicaid application form. The test score is calculated as the sum of correct answers to 36 STOFHLA questions, ranging from 0 to 36, to which the respondents scoring 23-36 are regarded as having adequate health literacy level in most healthcare setting.

b) The Newest Vital Sign (NVS) Health Literacy Assessment (timepoint 3) module assessed the respondents' ability to correctly communicate health information given general medical guidelines. The module verbally asked the respondents to answer six NVS questions regarding the information provided in an ice-cream container's nutrition label [3].

(3) ***Behavioral Phenotypes -*** BIG 5 Personality traits

Personality module administered the Five-Factor Model of Personality inventory test [10] to the respondents in 1992-1993 collection wave: *extraversion, openness, neuroticism, conscientiousness, and agreeableness.* The respondents indicated whether they agreed with each given descriptive statement of five characteristic, using a scale ranging from 1 (strongly disagree) to 6 (strongly agree). Mean summary scores for each characteristic were calculated and provided, and ranged from 2 to 12.

(4) ***Behavioral Phenotypes –*** Leisure Activity

In an aim to find health or social implications, we investigated leisure time activities of the WLS respondents in order to examine the genetic effects of cognition regarding how time is allocated in individual habits, social roles, and behavioral preferences. In timepoint2 of the survey, the contents of participants' leisure activities were self-reported in hours per week; (a) reading books, magazines, newspapers or other reading materials; (b) painting, drawing, or doing another form of art; (c) attendance to cultural events, such as going to a lecture, concert, play, museum or other similar activity; (d) going out to a restaurant or bar; (e) working on a crossword puzzle or other word game; (f) doing light physical activities that you do alone, such as light housework, gardening, or walking by yourself; (g) doing light physical activities that you do with others, such as walking with friends, bowling, playing softball or other team sports with light activity; (h) doing vigorous physical activities that you do alone, such as jogging, swimming,

biking, or going to the gym by yourself; (i) doing do vigorous physical activities that you do with others such as playing team sports, jogging, swimming, biking, or going to the gym with friends; (j) writing letters (not including email); (k) watching movies, (l) do hand crafting or hobbies, (m) making home/car repairs/other hand works; (n) go fishing or hunting; (o) watch Televisions; (p) talking on the phone with friends or relatives. In order to cover some outliers with extreme hours of certain activity, we took the logarithm of the reported hours for each activity and ran linear regression with the built GPSs.

(5) Occupational Status Variables and Spouse IQ

a. Occupational Education Score: In round 5 of the survey (2003-2005), the WLS collected the first or only job of the respondents based on their current or last employment information. The measure assigned a numeric value to its industry or class-of-worker categories based on the 1990 US Census data, which represents a percentage of persons who had at least a year of college education, ranging from 0 to 999.

b. Occupational Income Score: For first or only job of the respondents in the current or last employment modules, the WLS assigned the 1990-basis occupational earning scores, which represent the percentage of persons in the 1990 US Census data in an industry or class-of-work category who earned more than $14.30/hour in 1989, ranging from 37 to 876.

c. Spouse IQ: Previous literature have suggested the psychiatric hypothesis of assortative mating in academic achievements and IQ [11-15]. As spouse IQ data were available through the additional panels of WLS, we assessed the behavioral genetics of spouse concordance for IQ. With the spouse IQ variable, the analysis will investigate if behaviors of assortative mating have pleiotropic associations with GPS of cognitive abilities.

References.

1 Wechsler D. The Wechsler Adult Intelligence Scale — Revised. The Psychological Corporation. 1981.

2 Wechsler D. WAIS-III, Wechsler Adult Intelligence Scale: Administration and scoring manual. 1997.

3 Hubbard RC. Newest Vital Sign (NVS). https://pfe-pfizercom-prod.s3.amazonaws.com/health/nvs_flipbook_english_final.pdf.

4 Maenner MJ, Greenberg JS, Mailick MR. Association Between Low IQ Scores and Early Mortality in Men and Women: Evidence From a Population-Based Cohort Study. 2015;120(3):244-57.

5 Hauser RM, Palloni A. Adolescent IQ and Survival in the Wisconsin Longitudinal Study. The Journals of Gerontology: Series B. 2011;66B(suppl_1):i91-i101.

6 Henmon V. (Boston: Houghton-Mifflin Company, 1946).

7 Henmon VACJC, Houghton-Mifflin. The Henmon-Nelson tests of mental ability. Manual for administration. 1954;171:297-318.

8 Henmon VAC, Holt FO. A Report on the Administration of Scholastic Aptitude Tests to 34,000 High School Seniors in Wisconsin in 1929 and 1930: Prepared for the Committee on Cooperation, Wisconsin Secondary Schools and Colleges. Bureau of Guidance and Records of the University of Wisconsin; 1931.

9 Willingham WW, Pollack JM, Lewis C. Grades and Test Scores: Accounting for Observed Differences. Journal of Educational Measurement. 2002;39(1):1-37.

10 John OP, Donahue EM, Kentle RL. (Berkeley, CA: University of California, Berkeley, Institute of Personality …, 1991).

11 Halpin B. Educational homogamy in Ireland and Britain: trends and patterns. Br J Sociol. 2003;54(4):473-96.

12 Mascie-Taylor CG, Vandenberg SG. Assortative mating for IQ and personality due to propinquity and personal preference. Behav Genet. 1988;18(3):339-45.

13 Watson D, Klohnen EC, Casillas A, Simms EN, Haig J, Berry DS. Match makers and deal breakers: analyses of assortative mating in newlywed couples. Journal of personality. 2004;72(5):1029-68.

14 Hur YM. Assortive mating for personaltiy traits, educational level, religious affiliation, height, weight, adn body mass index in parents of Korean twin sample. Twin research : the official journal of the International Society for Twin Studies. 2003;6(6):467-70.

15 Pan Y, Wang, K. Spousal concordance in academic achievements and IQ a principal component analysis. Open Journal of Psychiatry. 2011(1):15-19.
